# Prevention of Puerperal Sepsis

**Published:** 1892-08

**Authors:** 


BUFFALO MEDICAL AND SURGICAL JOURNAL.

A MONTHLY REVIEW OF MEDICINE AND SURGERY.

*EDITORS:*

THOMAS LOTHROP, M. D. - - WM. WARREN POTTER, M. D.

All communications, whether of a literary or business character, should be addressed to the managing editor: 284 Franklin Street, Buffalo, N. Y.

Vol. XXXII. AUGUST, 1892. No. 1.

### PREVENTION OF PUERPERAL SEPSIS.

The application of prophylaxis can have no more useful place than in the lying-in chamber. It is here that an ounce of prevention is worth many pounds of cure. It is doubtful whether puerperal sepsis can be cured in every instance of its invasion, but it is almost certain that it can be prevented in ninety-nine cases out of a hundred. The absorbing interest which is manifesting on this subject is apparent when medical societies in cities, towns and hamlets are everywhere discussing the question with a degree of earnestness, intelligence, and liberality that is as encouraging as it is instructive.

There can be no doubt that a great responsibility rests upon the obstetrician of today, greater than at any period in modern times. He must contest the ground with the abdominal surgeon for a record, and especially must he contest the field with the maternity physician for as good work in the mansion, the villa, the cottage, and the tenement house. The family physician, who is likewise the obstetrician in the rural districts, must be able to show as good a record as his brother who practises in the courts and alleys of the populous city, or as he who has the advantage service in the lying-in hospitals. A few years ago the crowded maternity was considered about the worst place in which a woman could be confined. It was often necessary to close up such an institution when invaded with an "epidemic" of puerperal fever. Now such a thing as closing a maternity by reason of a large number of consecutive deaths from puerperal sepsis is unknown. In a thousand cases we hear of scarcely a death, and there is no such thing as an "epidemic" of puerperal fever.

We have said that much responsibility rests upon the shoulders of the family physician in the rural districts, and he must look to it that his cases are properly prepared for confinement by clean hands that are versed in all that is meant by the term "asepsis." When the general practitioner can be made to enforce the simplest rules of asepsis that he perfectly well understands, but is careless about enforcing, we shall soon hear almost nothing of puerperal fever or of ophthalmia neonatorum. It is not competent to plead lack of time or inconvenience in carrying out those simple details of cleanliness which are the foundation of prophylaxis in the lying-in chamber, and which must be insisted upon where human life is at stake, or loss of vision in the new-born is so often threatened.

